# Rheotaxis-based sperm separation using a biomimicry microfluidic device

**DOI:** 10.1038/s41598-021-97602-y

**Published:** 2021-09-15

**Authors:** Iman Ramazani Sarbandi, Ali Lesani, Mahdi Moghimi Zand, Reza Nosrati

**Affiliations:** 1 Small Medical Devices, BioMEMS & LoC Lab, Department of Mechanical Engineering, College of Engineering, University of Tehran, 14399-55961 Tehran, Iran; 2 Department of Mechanical and Aerospace Engineering, Monash University, 20 Research Way, Clayton, VIC 3800 Australia

**Keywords:** Biophysics, Mathematics and computing, Engineering, Biomedical engineering, Mechanical engineering

## Abstract

Sperm selection is crucial to assisted reproduction, influencing the success rate of the treatment cycle and offspring health. However, in the current clinical sperm selection practices, bypassing almost all the natural selection barriers is a major concern. Here, we present a biomimicry microfluidic method, inspired by the anatomy of the female reproductive tract, that separates motile sperm based on their rheotaxis behavior to swim against the flow into low shear rate regions. The device includes micropocket geometries that recall the oval-shaped microstructures of the female fallopian tube to create shear protected zones for sperm separation. Clinical tests with human samples indicate that the device is capable of isolating viable and highly motile sperm based on their rheotaxis responses, resulting in a separation efficiency of 100%. The device presents an automated alternative for the current sperm selection practices in assisted reproduction.

## Introduction

Infertility is on the rise worldwide, affecting almost 15% of the couples (over 48 million couples)^1,2^. In nearly half of the cases, male-related issues such as low sperm count or poor motility are responsible for the infertility issue^3,4^. Assisted reproductive technologies (ART) have been developed and refined over 40 years to treat infertility^4^. ARTs include intrauterine insemination (IUI), in vitro fertilization (IVF), and intracytoplasmic sperm injection (ICSI), in which a selected subpopulation of high-quality sperm is either introduced closer to the egg or injected directly into the egg to achieve fertilization^5^. However, one of the major concerns about ART, and ICSI, is that they bypass almost all the natural selection barriers that sperm encounter in vivo^5^.

The selection of high-quality sperm is crucial to infertility treatment. The success rate of the ART treatment cycle and embryo development rate are both directly correlated with the quality of selected sperm^6^. Swim-Up (SU) and Density Gradient Centrifugation (DGC) are the two most commonly used clinical methods for selecting high-quality sperm^7–9^. These methods are time-consuming, labor-intensive, highly subjective, and they introduce sperm DNA damage due to centrifugation while also resulting in relatively low recovery rates^6–9^. It is crucial to develop new methods for rapid, automated, and non-invasive selection of high-quality sperm by mimicking the natural selection process in vivo*,* ultimately improving the ART outcome.

Microfluidics has emerged as a rapid and automated platform for cell separation and studying gamete function^10,11^, capable of providing geometrically confined microenvironments that closely mimic the natural in vivo environment^12–14^. To find new selection methods, these platforms have been used to study sperm motion^15,16^, their interactions with micro geometries of the female reproductive tract^17,18^, and their migration behavior in response to external stimuli like fluid flow or chemical gradients^19–22^. Boundary-following behaviour^23–25^ and sperm tendency to cross laminar flow streamlines^26,27^ have been used in microfluidics to develop rapid yet straightforward selection strategies, mainly due to the absence of centrifugation force that may cause sperm DNA damage^28–30^.

**Figure 1:**
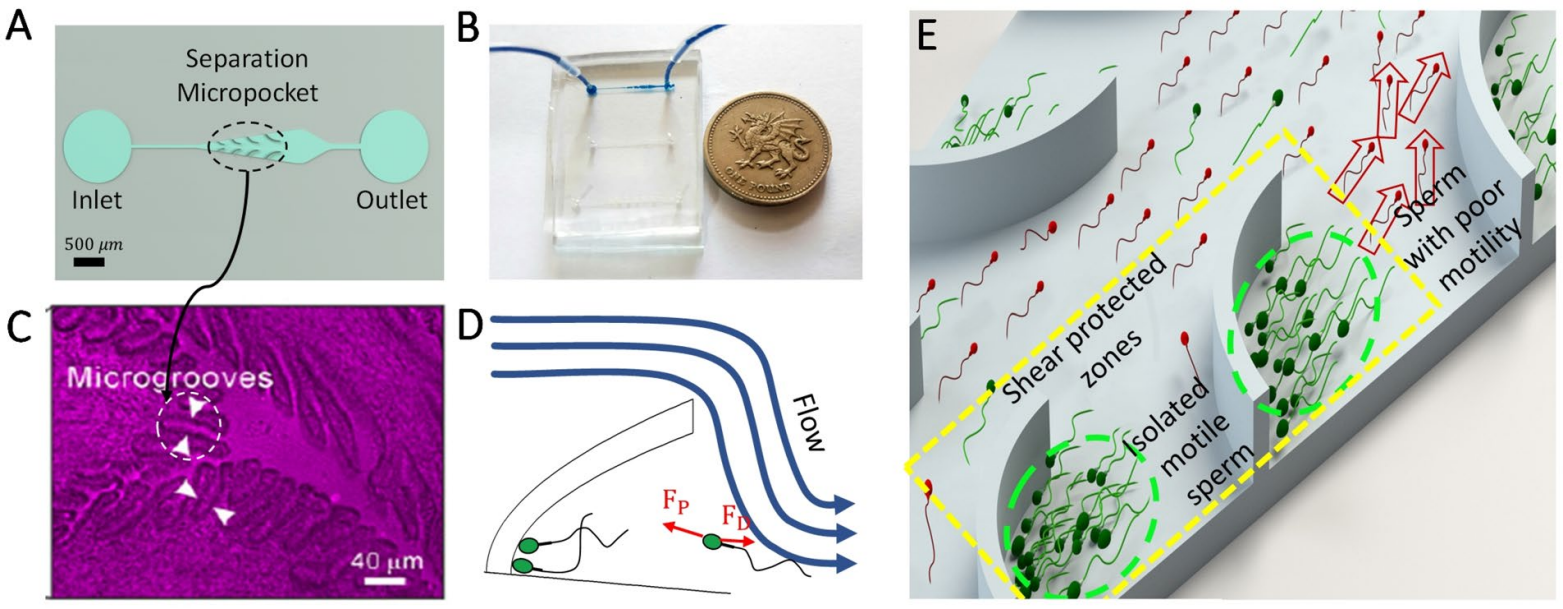
Microfluidic device featuring eight oval-shaped micro pockets for sperm separation. (**A**) Schematic view of the device geometry informed by oval-shaped microgrooves. (**B**) Photograph of the fabricated device. (**C**) the oval-shaped microgrooves within the female fallopian tubes as opposed to a part of the proposed microfluidic device^13^. (**D**) The rheotaxis-based separation process that reorients motile sperm against the flow to (**E**) isolate them into the selection micropockets. The sperm propulsive force, F_P_, should overcome the viscous induced drag, F_D_, to enable rheotaxis. Live and motile sperm (green) that exhibit rheotactic behavior were separated from the main flow, accumulating in the micropockets, while dead and non-motile sperm (red) were washed out of the device by the flow. Part C is reprinted with permission of the Proceedings of the National Academy of Sciences (PNAS), the official journal of the National Academy of Sciences (NAS)^13^.

Rheotaxis is one of the main guidance mechanisms in the reproductive tract, where the sperm cell reorients parallel to and swim upstream against the flow, towards the egg^31–33^. Due to the unique capability of microfluidics in controlling and manipulating fluidic microenvironments, rheotaxis has been used in microfluidics to isolate motile sperm^34^, including recent developments with diffuser type micro geometries that create favorable low shear rate zones to select motile sperm^35,36^. Also, recent approaches have tried to capture the physical aspects of the female tract geometry, but still, more efforts are needed to mimic the existing physical phenomenon. Here, we present a physiologically relevant approach, inspired by the mucosal flow through the oval-shaped microgrooves in the female fallopian tube, for rheotaxis-based separation of highly motile sperm (Fig. 1). Micropocket geometries in the device create shear protected zones along the microchannel walls to guide and isolate sperm. The finite element method (FEM) was used to simulate the velocity and shear rate distribution in the device. The device was fabricated and tested with human samples, and the results show that all the selected sperms were motile (100% efficiency). The results highlight the role of the female fallopian tube micro geometries on regulating shear rates in vivo to encourage rheotaxis-based navigation.

### The mechanism of rheotaxis-based sperm separation

Rheotaxis provides a long-range guidance mechanism for sperm to navigate through the female reproductive tract^31,37^**.** In a flow environment, the fluid flow imposes drag and rotational torques on both the sperm head and the flagellar envelop to reorient the sperm to swim upstream against the flow^31^**.** However, both the strength of the flow (and subsequent drag) and sperm motility level (i.e., propulsive force) contribute to the ability of sperm to exhibit rheotactic behavior. Specifically, at relatively low flow velocities, the propulsive force produced by the sperm flagellum is strong enough to overcome the flow-induced drag for upstream migration (Fig. 1D), while only strong enough flow can impose sufficient drag/torque to reorient the cell against the flow^13,32^**.** However, at relatively high flow velocities (high shear rates), the flow is strong enough to wash out the cells^33^**.** For human sperm, shear rates of 2 to 5 s^−1^ and flow velocities of 22 µm/s to 102 µm/s have been shown to encourage rheotaxis behaviour^13,19,38^. Inspired by the oval-shaped microgrooves in the female reproductive tract^13^ (Fig. 1C), the proposed device with micro geometries on the two side walls is shown in Fig. 1A. These micropockets suddenly expand the flow streamlines to create a sharp shear gradient in their frontal zone, facilitating sperm rheotaxis and a shear protected region inside the pockets for trapping and isolating motile sperm (Fig. 1E).

## Results and discussion

**Figure 2:**
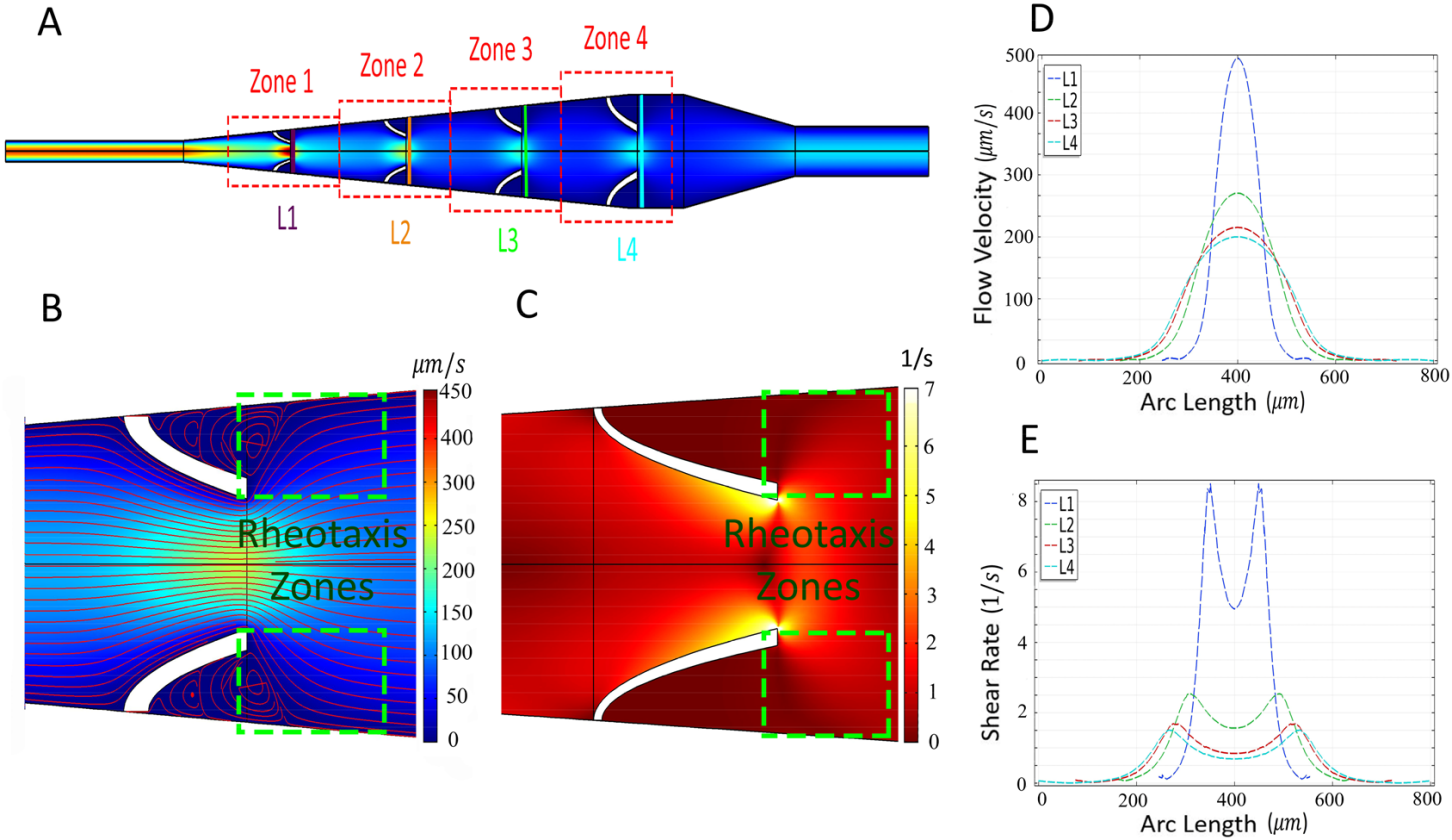
Finite element method simulation results of velocity field and shear rate distribution in the rheotaxis-based sperm separation device at a flow rate of 2 µl/min. (**A**) Velocity field in the device and (**B**) next to the micropockets in Zone 2. Red lines indicate flow streamlines. (**C**) shear rate distribution in Zone 2, indicating a favourable shear rate for sperm rheotaxis near the micropockets. (**D**) Velocity profile and (**E**) shear rate across the microchannel width in each zone and along the lines shown in (**A**).

To ensure the optimal performance of the device for rheotaxis-based separation of motile sperm, creating a gentle flow in frontal zones of the micropockets that encourages the upstream swimming behavior of motile sperm is essential. Hence, the injection rate of the sperm medium should be precisely estimated. The finite element method (FEM) simulation was performed to model sperm medium flow velocity, streamlines, and shear rate distribution in the device at a flow rate of 2 µl/min (Fig. 2). Due to the no-slip boundary condition on the walls, the flow velocity dropped across the microchannel width from a maximum value of ~ 450 $\mathrm{\mu m}/\mathrm{s}$ at the center of the channel to zero on the walls (Fig. 2A,B,D). This velocity gradient, together with the diffuser-like geometry of the microchannel, resulted in regions of low shear rates at the entrance to each of the four pairs of selection zones with an average shear rate of less than 3.2 s^−1^ (Fig. 2C,E). The average shear rate of less than 3.2 s^−1^ in each of the rheotaxis zones was used to ensure rheotaxis-based selection of motile sperm. To experimentally test the device, the device was tested with human sperm samples, and motile sperm were able to cross the flow streamlines and swim against the flow to enter the micropockets (Fig. 2B). This process enabled the selection of live and motile sperm in the micropockets while dead and immotile sperm are washed out of the device by the flow (Fig. 1E).

### Rheotaxis-based human sperm separation

**Figure 3:**
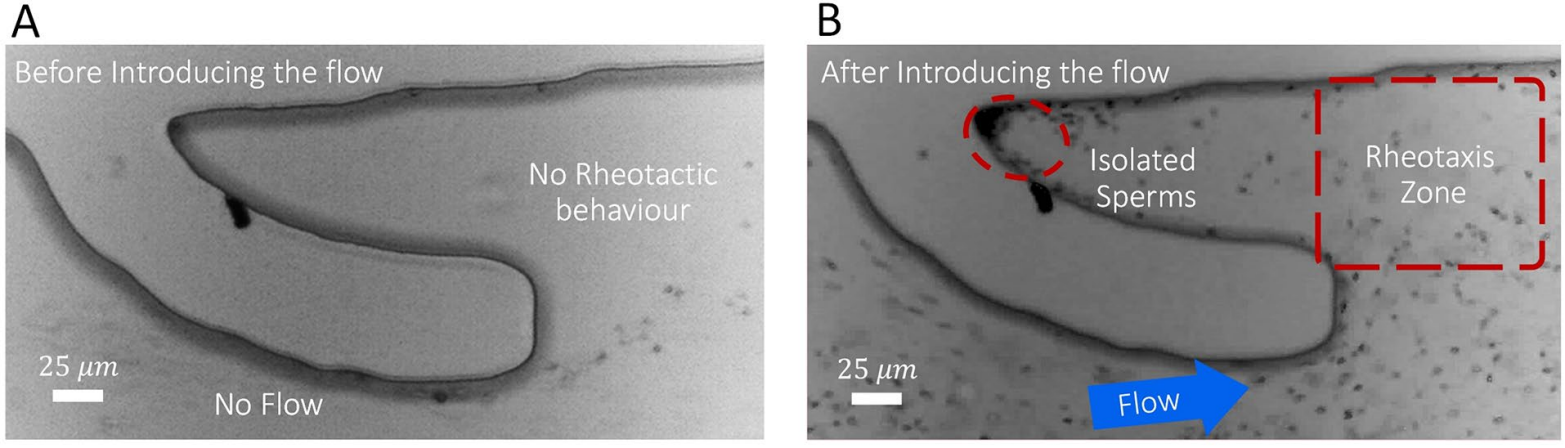
Rheotaxis-based isolation of sperm into micropockets. (**A**) In the absence of flow, sperm depart from the micropocket wall to follow the main microchannel’s wall towards the device outlet. (**B**) At a flow rate of 2 µL/min, motile sperm exhibited rheotaxis behaviour to enter the micropockets. The red dashed box indicates the shear protected zone in the frontal section of the micropocket.

To evaluate the performance of the device for sperm separation, the device was tested using fresh human samples. In the absence of flow (Fig. 3A), the majority of motile sperm exhibited boundary-following behavior to swim along the microchannel wall and then departed from the curved wall of the micropockets to continue swimming along the flat wall of the microchannel without entering the micropockets. In the absence of flow, the cells depart from the wall due to the sharp turn of the boundary into the micropockets^39^. However, by introducing an average flow rate of 2 µL/min (Fig. 3B), motile sperm were observed to reorient and swim across the flow to enter the shear protected zones inside the micropockets, with a relatively high density of responsive cells accumulating in the micropocket’s corners. At flow rates higher than 10 µL/min, the resulting shear rate in the rheotaxis zones was too high for sperm to exhibit the rheotaxis behavior, and the cells were advected downstream by the flow.

**Figure 4:**
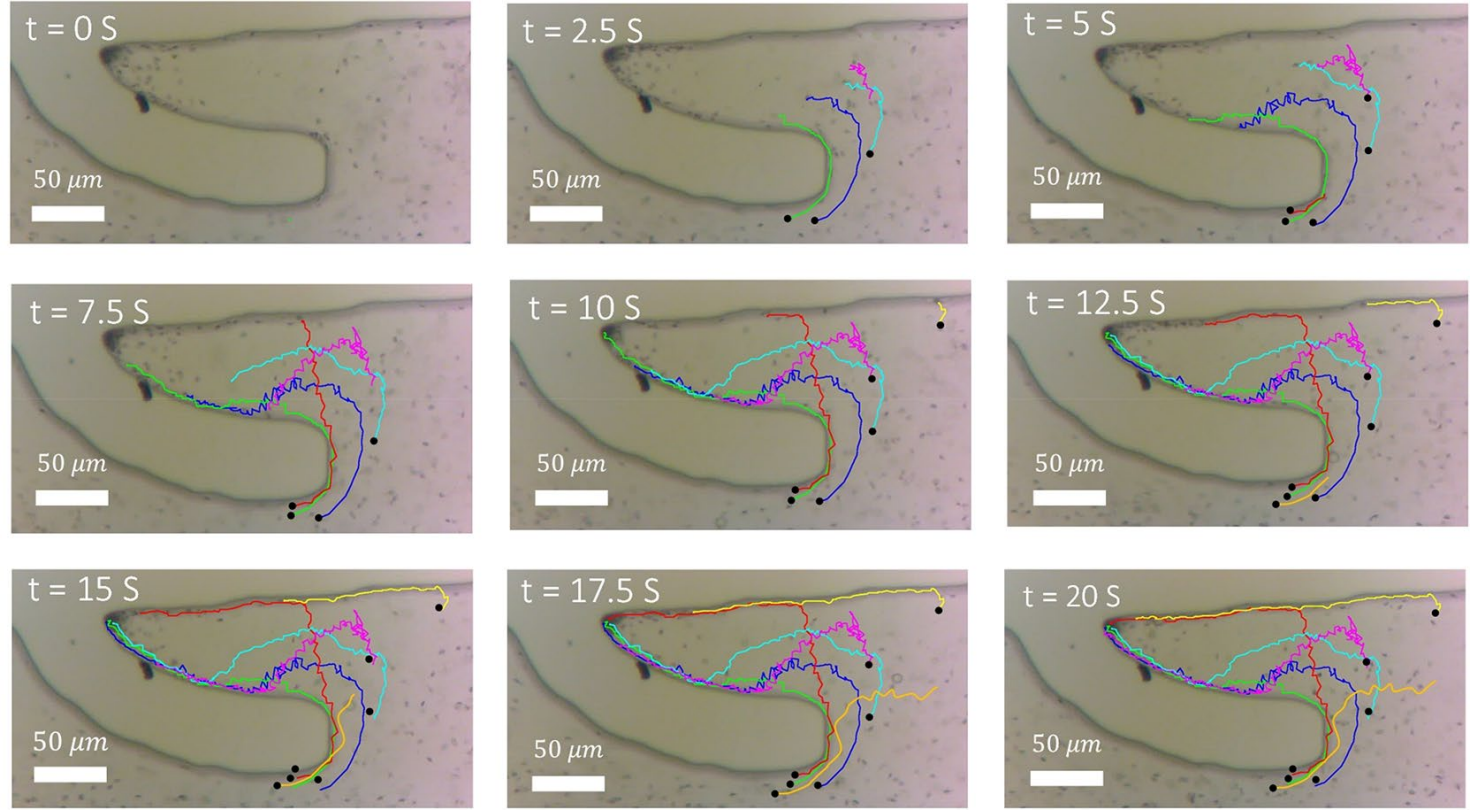
Image sequences of human sperm in the device, indicating the rheotaxis behaviour of motile sperm to swim into a representative micropocket. Swimming trajectories of 7 different sperm are depicted, with the trajectories starting at the solid black circles.

To demonstrate the rheotactic behavior of sperm in the device, sperm motion in the device and next to each of the separation micropockets were imaged for 1 min at 16 frames per second (see Video S1 and S2). The extracted trajectories of sperm near a micropocket in Zone 2 of the device are shown in Fig. 4, demonstrating that motile human sperm swim against the flow to enter the separation zones at a rate of at least 4.3 sperm per second. The interaction between the conical envelope of the flagellar wave and flow orients the cell in the upstream direction^35^ to direct the motile sperm to swim against the flow towards the low shear zones in the micropockets. It is noteworthy that only sufficiently motile sperm were able to exhibit rheotaxis behavior to overcome the drag and enter the micropockets, whereas less motile or dead sperm and debris were advected downstream by the flow.

After entering the micropockets, 68% of sperm were observed to follow the curved wall of the micropocket to accumulate in the corner (Fig. 3B). The remaining 32% of the tracked cells were observed to depart from the curved wall and then turned into the micropockets after reaching the straight wall of the main microchannel. Around 50% of sperm isolated in the micropockets were also observed to follow the boundary and swim back into the mainstream. However, since the number of sperm entering the micropockets was two-folds higher than the number of sperm leaving them, the net number of sperm isolated in each of the micropockets was always positive and increasing over time. Due to the rheotaxis separation process, as long as we have the flow, the number of entering sperm would be higher. Although the micropockets might also saturate to reduce this number, that is a prolonged process and needs several hours. The isolated motile sperm from the device was retrieved by replacing the buffer and then using a flow-off flow-on process. In this process, the raw sample in the main microchannel was replaced with a clean buffer, and then in the absence of flow, isolated motile sperm swim out of the micropockets into the main channel to be washed out of the device using a relatively slow flow (0.5 µL/min).

**Figure 5:**
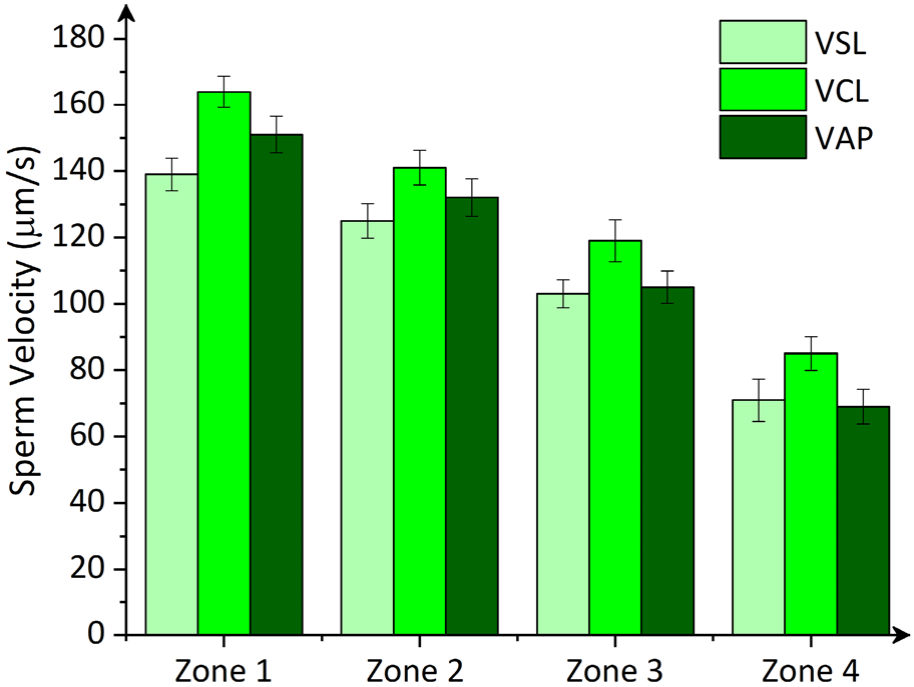
Straight line velocity (VSL), curvilinear velocity (VCL) and average path velocity (VAP) of human sperm isolated in each separation zone. Values are reported as mean ± s.d. (n ≥ 20).

Figure 5 compares the motility parameters for sperm isolated in each of the separation zones in the device. The results demonstrate that the device selects progressively motile sperm with an average curvilinear velocity (VCL) of 128 µm/s and corresponding average path velocity (VAP) and straight-line velocity (VSL) values of 115 µm/s and 110 µm/s, respectively (averaged over four zones). The fastest subpopulation of sperm with VCL of 164 µm/s, VAP of 151 µm/s and VSL of 140 µm/s were isolated in Zone 1, and then VCL, VAP, and VSL on average decreased by 19%, 23%, and 20%, respectively, for sperm isolated in each of the subsequent zones. The selection of the fastest sperm in Zone 1 is attributed to the higher shear rate experienced by the cells in this zone that only allows for highly motile sperm to overcome the flow and exhibit rheotaxis to enter the micropockets.

**Figure 6:**
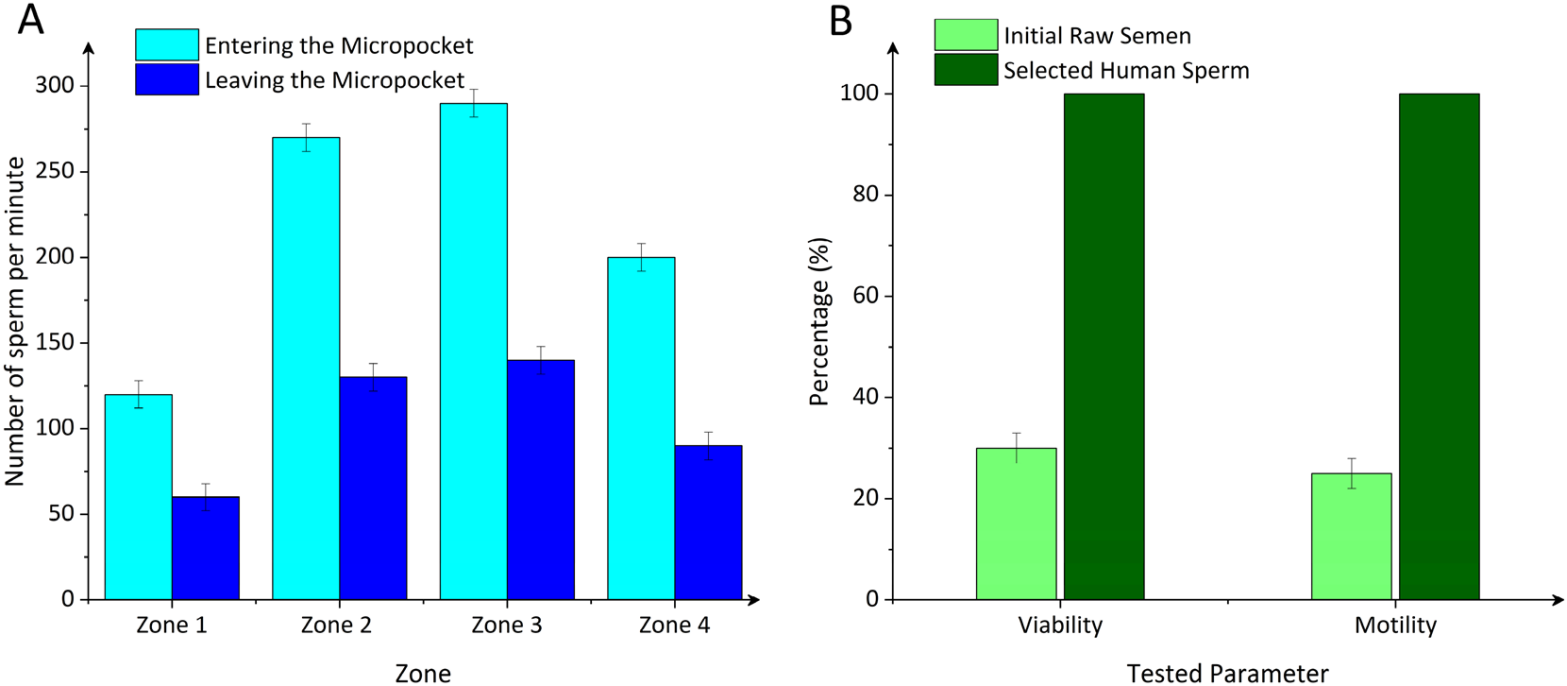
Selected sperm quality analysis at a flow rate of 2 µL/min. (**A**) Number of sperm entering and leaving the micropockets per minute as a function of selection zone. (**B**) Selected human sperm viability and motility as compared to the initial raw sample. Values are reported as mean ± s.d. (n ≥ 6).

Figure 6A shows the rate of sperm isolation in each separation zone. At a flow rate of 2 µL/min, motile sperm entered the micropockets in Zone 1 at a rate of 120 cells per minute (cell/min), and isolated sperm left the micropockets to return to the main channel at a rate of 60 cell/min, resulting in the net accumulation rate of 60 cell/min. A similar trend was also observed for sperm isolated in the other three zones (Zone 2, 3, and 4), with the number of sperm entering the micropocket (at a corresponding rate of 270, 290, and 200 cell/min) being approximately two-folds higher than the number of sperm leaving it (at a corresponding rate of 130, 140 and 90 cell/min). The net rate of sperm accumulation in Zone 2 and Zone 3 increased to 140 and 150 cells/min, respectively, but decreased to 110 cells/min in Zone 4. The separation results demonstrate that Zone 3 with an average flow velocity of 135 µm/s and a shear rate of 2.2 s^−1^ resulted in the highest rate of sperm isolation in the micropockets. Hence, in the next generation, a device with a geometry that matches zone 3 would be favorable. The high isolation rate in zone 3 is mainly attributed to the higher shear rates in Zone 1, and Zone 2 (smaller cross-sectional area) compared to Zone 3 that only allowed for a small subpopulation of highly motile sperm to exhibit rheotaxis. However, in Zone 4, the share rate was relatively low to effectively encourage sperm rheotaxis, resulting in the net accumulation rate decreasing by 31% compared to Zone 3. Considering the average concentration of the initial raw samples tested (65 M/ml) and the 20 min run time of the device, the net percentage of sperm isolated in each zone was 1.6%, 3.6%, 3.8%, and 2.6% in Zone 1, 2, 3, and 4, respectively. Finally, the isolated motile sperm were retrieved from the device by replacing the buffer and then using a flow-off flow-on process, as explained before.

Figure 6B shows selected sperm viability and motility as compared with the initial raw semen sample. Sperm selected from the device demonstrated over 70% and 75% improvement in viability and motility. Viability and motility of sperm in the initial raw samples were assessed by the computer-aided sperm analysis (CASA) test to be 30% and 25%, respectively, and both increased to 100% for selected sperm. Concerning natural fertilization, our findings highlight the role of flow and curved lumens within the female reproductive tract to provide low shear rate regions for rheotaxis-based selection of a subpopulation of highly motile sperm for fertilization. The device also provides new opportunities for the selection of high-quality sperm for assisted reproduction.

## Conclusions

In this work inspired by the anatomy of the female reproductive tract, we present a microfluidic device for rheotaxis-based selection of motile sperm based on their response to flow environments. The geometry of selection micropockets in the device is informed by the oval-shaped geometry of the microgrooves in the female fallopian tube that create shear protected zones for sperm guidance. By testing the device with human clinical samples, we demonstrate that the device is capable of isolating high-quality sperm with 100% efficiency (100% motility and 100% viability), which indicates over 70% improvement in quality as compared with the initial raw sample. Taken together, this rheotaxis-based sperm selection technology provides a simple and automated opportunity for the selection of highly motile and viable sperm in assisted reproduction.

## Method

### Computer simulation

The device geometry was imported into COMSOL Multiphysics (version 5.3a). The Navier–Stokes (Eq. 1) and conservation of mass (Eq. 2) equations were solved for 3-dimensional flow in a laminar flow module with no-slip boundary condition on the walls. The simulations were performed for 20 input flow rates between 0.1 μL min^−1^ and 2 μL min^−1^ at steps of 0.1 μL min^−1^ and for 10 input flow rates between 2 μL min^−1^ and 12 μL min^−1^ at steps of 1 μL min^−1^:

1 $$\uprho \left(\mathrm{V}.\nabla \right)\mathrm{V}= -\nabla \mathrm{P}+\upmu ({\nabla }^{2}\mathrm{ V})$$

2 $$\nabla .\mathrm{V}=0$$

where **V** is velocity, ${\varvec{\rho}}$ is density, ${\varvec{\mu}}$ is dynamic viscosity, and P is pressure.

### Device fabrication

The device was fabricated using conventional soft lithography techniques^36^. The device design comprises a 1.5 mm-long microchannel (100 $\mathrm{\mu m}$ in depth) with a diffuser section that include three pairs of micropockets ranging in radius of curvature from 150 to 300 $\mathrm{\mu m}$. SU-8 negative photoresist (Sigma, USA) was spin-coated on a silicon wafer. Afterwards, a designed photomask was used to selectively cure the coated SU-8 layer using ultraviolet (UV) light, forming the channels. The resulting structures were then used as a mold to cast polydimethylsiloxane (PDMS). The PDMS was prepared by mixing Sylgard 184 (Dow Corning Co., USA) and its curing agent at a ratio of 10:1. The mixture was poured into the mold and left at 75 °C for 1 h. The PDMS channel structures were immersed in acetone in an ultrasonic bath for 5 min, rinsed with deionized water, and then dipped in 10% H_2_SO_4_ solution for 30 min. After punching the inlet and outlet holes, the PDMS and glass were then exposed to oxygen plasma for 30 s and 300 s, respectively, and bonded to one another, forming the complete device geometry.

### Semen sample preparation

The human semen samples were provided by Avicenna Infertility Clinic. All participants signed informed consent, and the study was approved by the ethical Committee of Avicenna Research Institute (ACECR-AVICENNA-REC-1398–028). Six fresh human semen samples with an average concentration of 65 × 10^6^ cells per ml, the viability of 30%, and motility of 25% were used in our experiments. All the sperm parameters were assessed using the CASA test performed by the Avicenna Infertility Clinic. Collected raw semen specimens were first liquefied at 37° C for 30 min.

### Experimental procedure

At first, the device was filled by submerging it in buffer and applying vacuum pressure (− 30 psi) for 30 min and then incubated for about 1 h at 37 °C until use. The device was first placed on an inverted Olympus CK2 optical microscope. Two identical tubes were glued to the inlet and outlet ports to avoid leakage. A syringe pump (TYD01-01 Lead Fluid, China) was used to flow the sperm sample and buffer on-chip. The device was washed by flowing Ham’s F10 medium through the inlet. The prepared semen sample was then introduced on-chip using the syringe pump at a set flow rate of 1 to 10 µL/min for 20 min; during this time, motile sperm swim out of the mainstream into micropockets. A portable camera (Eyescope, NXM-EPA200) mounted on the microscope eyepiece was used to capture image sequences of sperm in the device at 10 × magnification at 16 frames per second for 20 min. The captured videos were analyzed in ImageJ (version 1.51j8; NIH) to manually track sperm head locations (20 sperm were tracked per each micropocket) and extract motility parameters, including curvilinear velocity (VCL: the distance transversed by sperm between every two consecutive frames divided by the time difference), straight-line velocity (VSL: the distance between the first and the last sperm position in each trajectory divided by the total time) and average path velocity (VAP: the time-average velocity of a sperm head along its average path). Moreover, the numbers of sperm entering and leaving the pockets were counted manually in ImageJ. After about 20 min, the sperm retrieval process was performed by a flow-off flow-on process, where the main flow was cut off for about 5 min to let the isolated motile sperm swim out of the micropockets into the main channel and then to be washed out of the device using a relatively slow flow (0.5 µL/min).

## Additional information

In this research, the human semen samples were provided by Avicenna Infertility Clinic. All participants signed informed consent, and the study was approved by the ethical Committee of Avicenna Research Institute (ACECR-AVICENNA-REC-1398–028). Also, the authors confirm that all experiments were performed following relevant guidelines and regulations.

## Supplementary Information

Supplementary Legends.

Supplementary Video 1.

Supplementary Video 2.
